# Current Concepts and Future Directions for the Assessment of Autoantibodies to Cellular Antigens Referred to as Anti-Nuclear Antibodies

**DOI:** 10.1155/2014/315179

**Published:** 2014-04-27

**Authors:** Michael Mahler, Pier-Luigi Meroni, Xavier Bossuyt, Marvin J. Fritzler

**Affiliations:** ^1^INOVA Diagnostics, Inc., 9900 Old Grove Road, San Diego, CA 92131-1638, USA; ^2^Rheumatology & Experimental Laboratory of Immuno-rheumatology, University of Milan, Istituto Auxologico Italiano, Via G. Zucchi 18, 20095 Cusano Milanino, Milan, Italy; ^3^Department of Microbiology and Immunology, Laboratory Medicine, University Hospitals Leuven, KU Leuven, Belgium; ^4^Faculty of Medicine, University of Calgary, Calgary, Alberta, Canada T2N 4N1

## Abstract

The detection of autoantibodies that target intracellular antigens, commonly termed anti-nuclear antibodies (ANA), is a serological hallmark in the diagnosis of systemic autoimmune rheumatic diseases (SARD). Different methods are available for detection of ANA and all bearing their own advantages and limitations. Most laboratories use the indirect immunofluorescence (IIF) assay based on HEp-2 cell substrates. Due to the subjectivity of this diagnostic platform, automated digital reading systems have been developed during the last decade. In addition, solid phase immunoassays using well characterized antigens have gained widespread adoption in high throughput laboratories due to their ease of use and open automation. Despite all the advances in the field of ANA detection and its contribution to the diagnosis of SARD, significant challenges persist. This review provides a comprehensive overview of the current status on ANA testing including automated IIF reading systems and solid phase assays and suggests an approach to interpretation of results and discusses meeting the problems of assay standardization and other persistent challenges.

## 1. Introduction

In 1950, Coons and Kaplan described the improvement of an immunofluorescence method for the detection of antigens in tissue cells [1]. Eight years later, Friou et al. first described an indirect immunofluorescence (IIF) assay for the detection of anti-nuclear antibodies (ANA) [2, 3]. Along with the earlier discovery of the lupus erythematosus (LE) cell and the development of the LE cell test [4, 5], this ushered in a long and productive age of ANA testing. The ANA IIF test initially relied on rodent tissue substrates but contemporary tests use HEp-2 cells, a cell line established in 1952 by Moore and her colleagues from tumors that had been produced in weanling rats exposed to irradiation and corticosteroids injected with epidermoid carcinoma tissue from the larynx of a 56-year-old male [6]. In the following decades, ANA tests using HEp-2 cells revolutionized the diagnosis of ANA associated rheumatic diseases (AARD) including systemic lupus erythematosus (SLE), systemic sclerosis (SSc), Sjögren's syndrome (SjS), mixed connective tissue disease (MCTD), and idiopathic inflammatory myopathies (IIM) [7, 8].

The IIF assay on HEp-2 cells has been replaced in many laboratories since the development of ANA screening assays based on ELISA and automated, high throughput multiplex assays using addressable laser bead and other array technologies for the detection of specific ANA [9, 10]. Due to a significant prevalence of “false negative” ANA results on these newer platforms and an insufficient communication between laboratorians and clinicians, there have been growing concerns about unilateral adoption of these newer screening and high throughput assays [11]. Questions about which method should be used and the lack of standardization of the novel test algorithms led the American College of Rheumatology (ACR) to form a task force who recommended the use of the conventional IIF HEp-2 platform for ANA detection [12]. This recommendation was, in part, based on evidence that the HEp-2 cell substrates are essentially an “array” presenting >100 autoantibody targets whereas most high throughput screening arrays are much more limited in autoantibody target composition. This has prompted a reevaluation of the ANA IIF method which was reflected by entire sessions dedicated to HEp-2 ANA testing at international clinical and scientific meetings.

In recent years, the first digital imaging systems for ANA IIF have been developed which eliminate some major drawbacks of the method, namely, the subjectivity of observers reading the slides and the lack of an automated procedure [15–17]. Nevertheless, several drawbacks of the HEp-2 IIF methods persist and other technologies for ANA detection continue to emerge and evolve. In this review, novel insights and updates on ANA detection are presented and the pros and cons of different methods are discussed.

## 2. Statistical Considerations

### 2.1. Sensitivity and Specificity

For diagnostic applications, it is important to differentiate between analytical sensitivity/specificity and clinical (diagnostic) sensitivity/specificity. Therefore, the terms clinical sensitivity and specificity, false negative, false positive, and predictive values are described in Table 1. In addition, it is widely known and extensively documented that certain autoantibodies can precede the diagnosis or full clinical expression of an underlying disease for many years and thus false positive results at a given point in time might, over a subsequent time period, become a true positive [18, 19]. Consequently, the term “false positive” for autoantibodies needs to be used carefully.

### 2.2. ROC Analysis and Cut-Off Selection

The receiver operating characteristic (ROC) analysis has a broad range of applications and was first used for military purposes during World War II [20]. In medicine, ROC analysis has been extensively used for diagnostic testing to evaluate the effectiveness of a novel diagnostic method as compared to an already established one or so called “gold standard” method. Several statistical methods can be applied using the ROC analysis including the most commonly used area under the curve (AUC). The AUC is equivalent to the Mann-Whitney *U*, which tests for the median difference between scores obtained in the two continuous data sets. However, any attempt to summarize the ROC curve into a single number fails as information about the pattern of tradeoffs of the particular discriminator algorithm is not expressed.

The manner by which immunoassay cut-offs are established varies significantly among researchers and scientists in diagnostic companies. A common approach to define the cut-off value for certain assays is to test specimens from patients with the respective disease and compare them to a broad range of controls including related and unrelated diseases as well as age and gender matched (apparently) healthy individuals. The mean value plus 3-fold standard deviation, the 95% or the 99% percentile, of the controls are then often used to define the cut-off value. Another popular approach for definition of the cut-off value makes use of ROC analysis. Despite broad application, most references do not specify how to use the ROC analysis to define the cut-off value [21]. In the majority of cases, a visual approach is used to identify an appropriate point on the ROC curve which provides a good combination of sensitivity and specificity. Following this, it is important that the cohort used to define the cut-off (training set) is large enough to achieve statistical power and that the cut-off is validated using an independent cohort of patients (validation set).

The method used for cut-off definition strongly depends on the assay and how it is intended to be used in a routine setting. For a screening assay, a high degree of sensitivity is mandatory to ensure that the number of patients that are missed by the assay is kept as low as possible. In contrast, confirmation tests need high specificity. In general, low cut-off values increase the sensitivity at the expense of decreasing specificity and* vice versa* when a higher cut-off is defined [9, 10].

The interpretation of the ANA test results depends on the pretest probability of having the disease or whether fulminant disease is present. In a setting of high index of suspicion, even low titers of ANA-IIF can be interpreted as significant [22]. In 1997 it was suggested by Tan and colleagues [23] that the test should be performed and reported at two dilutions, 1 : 40 and 1 : 160, in order to preserve the appropriate sensitivity and specificity. A more recent study recommended using a more economical single screening dilution of 1 : 160 [24] which was confirmed by a recommendation paper that used a Delphi approach for assessment of autoantibodies to intracellular antigens [21]. ANA-IIF testing on HEp-2 cells is currently widely accepted as the procedure of choice for the detection of ANA but not as a corollary of disease activity or relapse [21].

### 2.3. Likelihood Ratio

In clinical practice, an important and relevant question is “What is the probability of a patient having a particular disease when the laboratory test is positive or not having the disease when the laboratory test is negative?” [25]. Clinicians and laboratory professionals have difficulties in estimating the posttest probability for a disease based on sensitivity and specificity (2). Likelihood ratio (LR) is an alternative, and probably more easily understood, way to convey diagnostic accuracy data in a clinical setting [26]. The LR for a disease is the probability of the test result in patients with the disease divided by the probability of the same test result in individuals without the disease. The posttest probability for disease associated with a particular test result can be estimated based on the pretest probability and the LR for that particular test result [25, 27].

Traditionally, a single cut-off is used for the interpretation of a laboratory test and all values above or below the cut-off value are given the same interpretation (positive or negative, resp.). For many AARD, the likelihood for disease increases with increasing antibody concentration [28–30]. This information is lacking when a single cut-off is used. LR can be assigned to a particular test result or to a test result interval (e.g., the antibody titer of ANA). It has been shown that the LR for an AARD increases with increasing antibody levels [28]. Knowledge of test result (interval) specific LR improves the clinical interpretation of a particular test result compared to knowledge related to a single cut-off value.

The LR gives an estimation of whether there will be a significant change in pretest to posttest probability of disease given the test result [25]. A LR of 1 implies that there will be no difference between pretest and posttest probability [25]. LRs >10 or <0.1 indicate large, often clinically significant, differences. LRs between 5 and 10 and between 0.1 and 0.2 indicate modest clinical differences [25].

**Figure 1:**
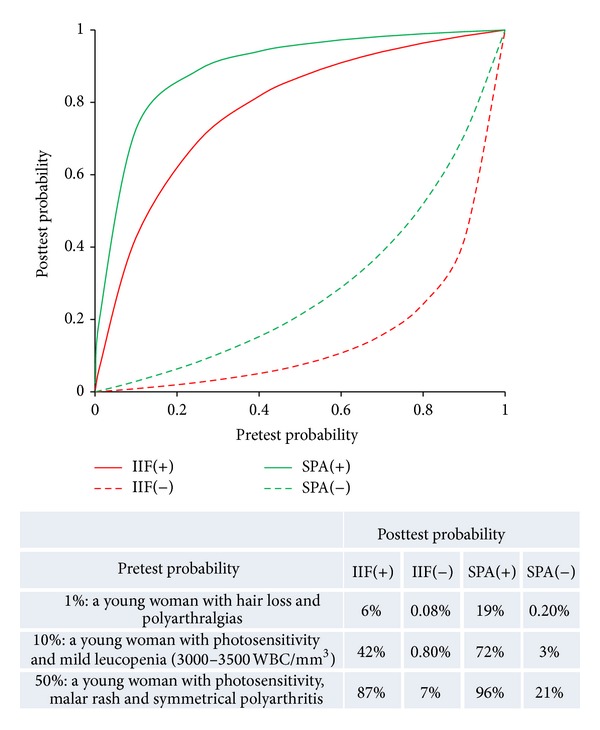
Illustration of pretest and posttest probability. Posttest probability (predictive value) for systemic lupus erythematosus as a function of pretest probability and as a function of indirect immunofluorescence (IIF) and solid phase assay (SPA) (EliA CTD screen, Thermo Fisher) test result. Values for likelihood ratios are from Bossuyt and Fieuws [31], WBC = white blood cell.

The LR for SLE for ANA by IIF has been estimated to be 7 for a positive test result and 0.03 for a negative test result, whereas the LR for SLE based on solid phase assays (SPA) [in this case: Fluoro enzyme immunoassay (FEIA), EliA CTD screen] has been estimated to be 24 for a positive test result and 0.27 for a negative test result [31]. Using LRs, one can calculate the posttest probability for any given pretest probability [25].  Figure 1 illustrates a graphical representation of the posttest probability (predictive value) for SLE as a function of the pretest probability for IIF as well as for SPA. Such graphical representation has been shown to be a convenient way to convey diagnostic information [26].

To illustrate the impact of pretest probability and assay performance on the posttest probability clinical examples are provided (unpublished data, based on expert experience of Pier-Luigi Meroni).

For example, a young woman with hair loss and polyarthralgias, which are very nonspecific signs of SLE, is estimated to have a pretest probability for SLE of 1%. If ANA by IIF turns out to be positive in this patient the probability for SLE increases from 1% to 6%, which is still low. If the SPA CTD screen reveals an autoantibody directed to a specific nuclear antigen (ENA or dsDNA), then the probability for SLE is higher (19%). A second example is a young woman who presents to her physician with photosensitivity and mild leucopenia (3000–3500 WBC/mm^3^). Based on this clinical presentation, the probability for SLE is estimated to be 10%. A positive ANA by IIF increases the probability for SLE to 42%, whereas a positive SPA CTD screen increases the probability for SLE to 72%, which makes the diagnosis for SLE likely. However, a woman positive for anti-SSA/Ro could display the same clinical manifestations not necessarily having SLE (sometimes the sicca syndrome is clinically silent at the beginning of a primary SjS). A negative ANA by IIF result would reduce the probability for SLE from 10% to <1%. The third example is a young woman with photosensitivity, malar rash, and symmetrical polyarthritis. This clinical picture is suggestive of SLE (50% probability). A positive IIF result increases the probability for SLE from 50% to 87%, whereas a positive SPA CTD screen increases it to 96%. A negative IIF result would reduce the probability for SLE to 7%, whereas a negative SPA test result would reduce the probability of SLE to 21%, illustrating the NPV of SPA is lower than the NPV of IIF.

## 3. Nomenclature of Antibodies to Cellular Antigens Commonly Referred to as Anti-Nuclear Antibodies (ANA) 

Historically, only antibodies targeting antigens present in the nuclear compartment of the cells (nuclear antigens) were called ANA. Similarly, the term extractable nuclear antigen (ENA), described in 1959 by Holman and Robbins was used for a group of nuclear antigens extractable by saline solutions [14]. Nowadays, with the identification of a variety of new autoantigens within various compartments of the cell, the nomenclature has become rather imprecise and misleading [21]. As an oversimplification, even serum samples with anticytoplasmic but without ANA reactivity are sometimes considered as ANA positive [32–34]. This confusing terminology has even been adopted in the nomenclature of commercial autoantibody assays and kits. Autoantibody arrays on various technology platforms are often termed as ANA or ENA profiles even though they contain relatively insoluble nuclear antigens such as dsDNA and/or cytoplasmic targets such as ribosomal P or Jo-1 antigens. Therefore, standardization of this nomenclature is highly desirable.

## 4. Anti-Nuclear Antibodies in Different Conditions

To date, more than 160 autoantigens, many of them localized to the cell nucleus, have been described in sera of SLE patients [35]. Therefore, the spectrum of SLE associated autoantigens contained in most ANA screening SPA includes only a small proportion of antigens targeted by SLE autoantibodies [35, 36]. However, most SLE associated autoantigens, apart from the standard ENAs, are rarely the target of individual SLE sera and even more uncommon without reactivity to any of the standard ENAs. For example, a recent study found a sensitivity of a SPA for SLE of 79% (in diagnostic samples) compared to a sensitivity of IIF at cut-off 1 : 160 of 90% [29]. Thus, the number of SLE patients having at least one clinically meaningful autoantibody that are missed by ANA SPA appears to be approximately 10%. In contrast, an even larger proportion of SSc patients have a negative test results when an ANA SPA is used [37]. Consequently, the clinical utility of novel assays for different AARD can be different and each new assay has to be validated in all AARD subgroups (SLE, SSc, MCTD, SjS, and IIM).

**Figure 2:**
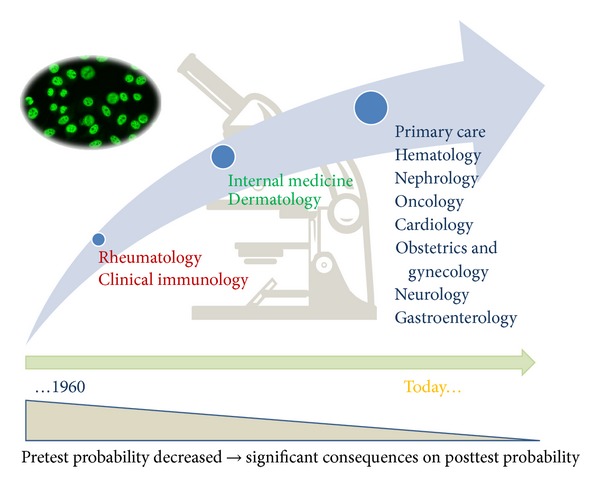
Change in referral patterns. Historically, when the ANA HEp-2 test became available in around 1960 exclusively rheumatologist and clinical immunologists ordered the ANA test. With the emerging recognition that many other diseases are associated with ANAs, a broad range of clinical disciplines order the ANA test. With changes in the ANA referral pattern and the associated decrease in the pretest probability, the posttest probability significantly decreases (indicated by the triangle).

In addition to SLE and SSc, ANA can be found in various other SARD including but not limited to IIM, SjS, and MCTD (Table 2). The appreciation that ANA are useful diagnostic biomarkers in a broad spectrum of autoimmune conditions has led to a significant change in the referral pattern of ANA tests to diagnostic laboratories (see Figure 1). Historically, primarily rheumatologists and clinical immunologists ordered ANA testing as an aid to the diagnosis of SLE. Much of this was due to the embedding of ANA and certain ENA in the older and now more recent classification criteria for SLE [38, 39]. Nowadays, a wider spectrum of clinicians order the ANA test (see Figure 1) including but not limited to internists, dermatologists, nephrologists, oncologists, cardiologists, neurologists, gastroenterologists, otolaryngologists, ophthalmologists, gynecologists, and even primary care physicians (Figure 2). This can be attributed to the broadening spectrum of ANAs in rheumatoid arthritis (RA), antiphospholipid syndrome (APS) [40], autoimmune liver diseases such as autoimmune hepatitis and primary biliary cirrhosis (PBC) [41–46], vasculitis [47], inflammatory bowel disease (IBD) [48–51], and cancer [52–57] (Table 4).

## 5. Differential Diagnosis of Autoimmune Diseases

The early and accurate diagnosis of autoimmune diseases can be very challenging because the spectrum of signs and symptoms are very wide and often overlap. Initially, an AARD has to be differentiated from a wide spectrum disorders (i.e., infections, malignancies, allergic, and adverse drug reactions) presenting with similar signs and symptoms. For example, a patient suspected to have SLE can first present with skin manifestations which need to be differentiated from discoid lupus, polymorphous light eruption, rosacea, drug eruptions, and other dermatoses. If other organs are involved (i.e., kidney, lung, musculoskeletal, cardiovascular, or neuropsychiatric [58]) the differential diagnosis must take other diagnostic possibilities into consideration. Secondly, after the presence of an AARD is confirmed on the basis of signs, symptoms, and physical examination, the different AARD need to be differentiated from each other so as to assist the clinician with decisions about appropriate therapeutic interventions. This can be further complicated by the evolution of autoimmune diseases from one condition to another. Many AARD, especially SLE, can present with arthritis but during follow-up, a diagnosis of RA or “rupus” might be established [59, 60]. Similarly, MCTD can evolve into SSc or RA. The appropriate interpretation of a positive or negative ANA can help enlighten the diagnostic and prognostic accuracy of AARD, although very little is known about the LR to differentiate the different diseases.

## 6. Screening and Profile Assays for ANA Detection

### 6.1. ANA by Indirect Immunofluorescence on HEp-2 Cells

For well over the last decade, the IIF HEp-2 assay was being replaced by newer technologies for the detection of ANA [61] and several larger laboratories switched to automated high-throughput immunoassay platforms [61]. However, in 2010, a position paper was published indicating that IIF on HEp-2 cells should remain the “gold standard” for the detection of ANA [12], triggering a renaissance of the IIF ANA test. Nevertheless, in some cases, an ANA result based on IIF ANA on HEp-2 substrates may mislead the clinician and has to be interpreted within the clinical context [62] (Table 5). In addition, standardization of this assay is difficult due to intermanufacturer variations in the substrate and the fixation process, characteristics of the secondary antibody used [63], interlaboratory variations in microscopy apparatus, and, especially, the subjective interpretation of the results [64]. Detection of ANA by IIF may also yield false negative results even in the presence of high titers of antibodies, such as those directed to SS-A/Ro60, Ro52/TRIM21, Jo-1 (histidyl tRNA synthetase), and others [65–67]. Additionally, the challenge of significant variation of staining patterns on the ANA HEp-2 IIF substrates obtained with slides from different manufacturers [63] has led to a proposed nomenclature for IIF patterns [13] (Table 6). For these reasons, considerable effort has been dedicated to the development of standardized SPA for routine use, such as ELISA [68], which are attended by guidelines for the detection of ANA [21, 62, 69].

#### 6.1.1. Automated Pattern Recognition of the ANA HEp-2 Test

Computer assisted pattern recognition for ANA testing on HEp-2 cells has been described more than ten years ago [70]. Automated hardware and software-based pattern recognition platforms that allow for the identification and archiving of IIF patterns obtained on HEp-2 cell substrates; however, they have only become available during the last few years [15, 16, 71–74]. The operating principle of these new automated systems is acquiring, storing, and analyzing of digital images of stained IIF slides and displaying them on high resolution computer monitors. The inherent technical difficulties of processing and reading IIF slides (manual reading, real-time interpretation, need for dark room, and handwritten results transcription) make traditional IIF methods difficult to fit in the workflow of modern, automated laboratories. The new automated systems are powerful workflow and operational tools that can eliminate the need for a darkroom and separate image acquisition from image interpretation and have the potential to improve the quality and utility of the ANA HEp-2 assay.

The currently available automated ANA IIF image analyzing systems include NOVA View (INOVA Diagnostics, San Diego, US) [30], Aklides (Medipan, Berlin, Germany) [15, 16, 75], G-Sight (Menarini, Florence, Italy) [76, 77], EuroPattern (Euroimmun, Lübeck, Germany) [73], Image Navigator (ImmunoConcepts, Sacramento, US), and Helios (Aesku, Wendelsheim, Germany) (Table 7). The systems differ from each other with respect to the use of DNA-binding counterstains, such as DAPI, the cell substrate used (e.g., most systems are restricted to using the respective manufacturer's slides), the throughput, the number of patterns that can be identified, and user-friendly features of the software [76, 77].

Generally, these automated systems are based on a microscope fitted with an automated stage, a CCD digital camera, a LED light source, and software that controls the moving parts and directs image acquisition. All systems perform some kind of fluorescent light intensity measurement and use the results for preliminarily categorization of the samples as positive or negative and for pattern analysis. The automated reading is followed by human visual interpretation of the digital images that are displayed on a computer monitor, allowing user confirmation or revision of the automated results. By providing good quality digital images and other objective information (such as preliminary classification and pattern interpretation), these automated systems support the operators' decision making and increase the consistency between readers and readings. In addition, the digital images can be stored for training, documentation, follow-up, and second opinion purposes. In the future, these digital images might also become part of the patient's electronic medical record (EMR). At present, the systems are highly reliable in their ability to discriminate positive from negative reactions and to estimate fluorescence intensity, but the accuracy and robustness of pattern recognition does not reach the accuracy of human interpretation [77, 78]. An important feature is, the quality of the digital images, enabling the operators to make the same clinically relevant interpretation as they would make using a conventional microscope.

The final result interpretation is made by the operator, therefore subjectivity cannot be completely removed. Moreover, the characteristics of the HEp-2 substrates and conjugates influence the appearance of certain ANA specificities, and the automated pattern identification of the various systems is likely based on somewhat different programming principals. Nevertheless, automated systems for ANA HEp-2 analysis are a significant step forward to reduce variability and offer opportunities to increase harmonization of ANA interpretation [79].

Some systems offer automated assessment of ANA endpoint titers on a single serum dilution, thereby eliminating the need for serial dilutions [75]. Moreover, the potential integration of the automated digital IIF systems with laboratory information systems (LIS) provides sample traceability, and eliminates manual transcription and associated transmittal errors, thereby improving patient safety. The systems also hold the promise to reduce hands on time. Work flow studies using different systems are required to analyze the efficiency benefits of those systems.

#### 6.1.2. Limited Sensitivity and Specificity of ANA HEp-2 IIF Test

Several studies have demonstrated limited analytical/clinical sensitivity [61, 63, 66, 80, 81] and clinical specificity [82–85], of IIF on HEp-2 cells. In particular for anti-Rib-P, anti-SSA/Ro60, and anti-Jo-1 autoantibodies, the ANA HEp-2 IIF test has been reported to lack analytical sensitivity which translates to the clinical sensitivity for AARD [63, 65, 66, 81, 86, 87]. In a comparative analysis of an ANA ELISA and ANA IIF, equivalent sensitivity but significantly higher specificity of the ELISA was observed [84]. When using the cut-off recommended by the manufacturer, the clinical specificity of IIF for AARD was as low as 62.3% [84].

Based on these observations, over three decades ago a transfected HEp-2 cell line overexpressing SS-A/Ro60 was developed and this cell-based IIF assay is marketed as HEp-2000 cells [81, 88–90]. A similar approach has recently been used for SmD1 antigen in a research HEp-2 cell line [91]. This technological approach of overexpressing target antigens in a variety of cell lines has more recently become a productive approach to cell based IIF assays where structural or conformational epitopes are important for human autoantibody detection. It was found that adding antigen specific assays to IIF on HEp-2 cells significantly improved the diagnostic algorithm for the diagnosis of SARD [92]. However, it was concluded in this study that changing from IIF to other methods for ANA detection also required modification of the disease criteria. In addition, it was highly recommended to use anti-SS-A antibody assays in addition to ANA HEp-2 test [92].

### 6.2. Different Staining Patterns Have Different Specificities

**Figure 3:**
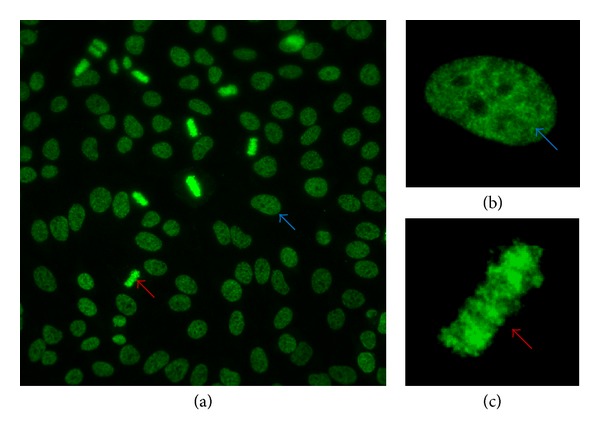
Characteristic staining pattern of anti-DFS70 antibodies. The characteristic dense fine speckled (DFS) staining pattern of interphase HEp-2 cells is indicated by the blue arrow and the strong chromatin staining of mitotic cells by the red arrow. (a) Wide field view using 40x magnification, (b) dense fine speckled pattern of an interphase nucleus, and (c) of the metaphase chromatin of a mitotic cell.

Disease classification criteria, such as the SLE criteria [38, 39], do not distinguish between different ANA IIF patterns. Although certain ANA patterns on HEp-2 cells have a significant disease associations, in clinical practice differentiation of IIF patterns is rarely used as an aid in establishing the clinical diagnosis [21]. Nevertheless, it is well established that the centromere staining pattern is primarily associated with the limited cutaneous form of SSc (also referred to as the CREST syndrome) [8, 93]. Additional examples are the association of the homogenous IIF pattern with SLE and nucleolar IIF pattern with SSc [37], although these generalizations have not been observed in all studies [94] (most likely attributed to different pretest probabilities). Interestingly, antibodies to dense fine speckled 70 (DFS70), also known as LEDGF (lens epithelium-derived growth factor), which generate a DFS IIF pattern on HEp-2 cells were not commonly observed in AARDs [82, 95–102]. These antibodies decorate interphase nucleoplasm outside of the nucleolus but in contrast to the anti-SSA/SSB and anti-Mi-2 antibodies, the anti-DFS70/LEDGF antibodies stain the metaphase and telophase chromosomal cell plates [13] (Figure 3). Just recently, it was confirmed that the DFS pattern was not associated with AARD and was primarily found in apparently healthy individuals [103]. In rare cases, this pattern can also be observed in patients with AARD, but these cases, anti-DFS70 antibodies are commonly accompanied by other autoantibodies [98, 102]. Follow-up of individuals with high titers of anti-DFS70 antibodies revealed that they retain ANA reactivity even after four years but do not develop SARD [103, 104]. Hence, it has been suggested that, within some limits, anti-DFS70 antibodies can be used to exclude the diagnosis of AARD [82]. The major epitope of the molecule is conformation dependent and is located in the C-terminal part of the molecule [105]. Although the immunoreactive region has been shown to be located within a stretch of 22 amino acids (407–435), the use of 12 mer peptides failed to establish reactivity with the presumed linear epitope [105].

### 6.3. ANA Screening ELISA

During the last decade, different strategies have been utilized to develop, evaluate, and commercialize several ANA screening ELISAs [106–110]. The majority of ANA screening ELISAs make use of mixtures or “blends” of purified autoantigens from native sources and/or recombinant technologies [106, 111]. The composition of these antigen preparations is quite diverse and is dependent on several factors including the availability of pure antigens and the technical feasibility of combining all different antigens in a single assay. Most available immunoassays contain SS-A/Ro60 [112], SS-B/La, Scl-70/topoisomerase I [113], CENP-B [93], Jo-1, U1-RNP, Sm, and dsDNA [114]. ANA screening ELISAs from some manufacturers also contain other autoantigens such as PM/Scl or ribosomal P [36]. However, based on comparison to immunoprecipitation of radiolabelled native proteins and other techniques, reactivity can be missed by these ELISAs even if the autoantigens are contained in the mixture [36]. This can be attributed to the notion that individual autoantigens exhibit different biochemical properties and, therefore, display different binding behaviors when solid phase matrices are used to bind the target autoantigens. For example, some antigens might bind to other targets in the same mixture resulting in a masking effect that gives the impression that insufficient epitopes are available for human autoantibody binding. Some antigens, such as PCNA [115, 116], RNA Pol III [117], or Th/To [118], are rarely included as purified antigen in screening assays.

### 6.4. Line Immunoassays

Line immunoassays (LIA) can basically be considered second generation dot-blot assays. A broad range of LIAs are available and they are typically used to confirm autoantibodies previously identified by HEp-2 ANA IIF or other screening immunoassays [119]. However, in some laboratories, these LIAs have also been used as a screening test for disease specific autoantibodies that are seen in SLE, SSc, IIM, paraneoplastic, and autoimmune liver diseases [120]. There have been recent advances in partially automating these multiplex LIAs making them somewhat more appealing to high throughput laboratory testing [121]. Despite their ease of use, LIAs have some drawbacks including the lack of sensitivity and specificity for certain autoantibodies [120, 122]. The antigen compositions of several are shown in Table 3.

### 6.5. Multiplex Bead-Based Assays

Multiplex assays based on the Luminex technology (Austin, Texas, USA) use addressable laser beads and are therefore often referred to as ALBIA (addressable laser bead immunoassays) [9]. Today, several commercial ALBIA kits are available for the detection of autoantibodies to a variety of autoantigens [83, 85, 123–130]. First generation ALBIAs showed polyreactivity which was caused by nonspecific binding to the beads [131]. Second generation assays showed significant reduced polyreactivity and thus higher specificity [131]. In 2007, a multiplex test for the detection of ANA was compared to ANA IIF and different ELISA assays. 7/87 (7.4%) of healthy donors were positive, 6/7 showed a speckled, and 1/7 a nucleolar staining pattern [85]. Similar to LIAs, the number of antigens and the antigen compositions of these bead-based arrays significantly vary and are shown in Table 3.

### 6.6. Other ANA Tests

Additional methods have been developed for automated ANA detection [84, 108, 132–139]. In 1999, a fully automated ANA screening assay (COBAS Core HEp2 ANA EIA; Roche Diagnostics, Mannheim, Germany) was developed and evaluated. The performance evaluation studies showed promising results but were inclusive in the conclusion. One study shows that the new assay is superior to the ANA IIF as analyzed by ROC analysis [138]. However, this finding could not be confirmed in a second independent analysis [133]. Six years later, the first-automated chemiluminescent immunoassay (CIA) for the detection of ANA (LIAISON ANA screen, DiaSorin) was evaluated in two centers yielding a good positive (79.5%) and negative agreement (91.2%) when the LIAISON ANA screen was compared to the ANA IIF test (Bio-Rad) [140]. A recent study using this assay showed ANA prevalence compatible with the expected values of the ANA IIF test [42]. Only moderate agreement was found between IIF and a multiplex assay based on the ALBIA. The majority of ANA IIF positive and multiplex ANA negative sera were also positive by an ANA ELISA utilizing nuclear extracts [129]. In addition, several other methods have been developed for ANA testing, but are not widely used in clinical practice. In 2009, a novel method for quantitative ANA measurement using near-infrared imaging was also described [141]. Furthermore, a novel microbead-based ELISA system using fluorescence-coded immobilized microbeads on the AKLIDIS system has been described [142]. Similar to the ALBIA, but using nanobarcodes for the bead identification, the Ultraplex system [143] was used to screen simultaneously for nine ANA autoantibodies, requiring significantly less labor and fewer reagents, with performance equivalent to existing gold-standard methods.

Several publications have described protein arrays on planar solid phase surfaces for the detection of autoantibodies to a wide range of viral proteins and autoantigens [144, 145] bound to a variety of surfaces [146]. However, these immunoassays are still not used in routine diagnostic laboratories.

More recently novel “ANA” screening assays have been developed on fully automated closed systems such as the Phadia (Thermo Fisher, Freiburg, Germany) or the BIO-FLASH System (INOVA, San Diego, USA) [147]. The EliA CTD Screen (Thermo Fisher) has been evaluated in several studies, two of which have been published in peer-reviewed journals [28, 148]. The first study showed satisfactory results for anti-ribosomal P, anti-PM/Scl, anti-Mi-2, and anti-PCNA antibodies. However, the sensitivity for anti-fibrillarin and anti-RNA Pol III antibodies was rather limited [148]. In the second study, the CTD screen was compared to the ANA HEp-2000 method [29]. Additionally, the QUANTA Flash CTD Screen Plus (on BIO-FLASH) was evaluated and shown to exhibit good sensitivity in different SARD [149]. However, further studies are needed to establish the clinical performance characteristics of the novel assays.

## 7. Quality Aspects, Standardization, and Reference Sera

For the development and quality assurance of autoantibody assays a broad range of international reference samples are mandatory [150, 151]. Consequently, an ANA and related autoantibody reference serum panel was established and is now available through the Center for Disease Control and Prevention in Atlanta, USA [152–155]. Initially, this panel of sera was used to standardize ANA IIF tests and to define staining patterns, but eventually, this panel was used to evaluate the performance of different autoantibody immunoassays [152]. In 2000, another ANA reference serum panel became available through the Association of Medical Laboratory Immunologists (AMLI) [156]. However, only a few studies used these samples for their investigations [157]. The first international standard for ANA became available in 1990 [158]. However, the number of international reference sera is limited and not all autoantibodies are represented by the available serum panels [150]. Furthermore, for screening assays, monospecific samples for each antigen are required to ensure the presence of all antigens in sufficient quantity and quality.

Different committees and organizations were formed who worked to achieve a better standardized approach to autoantibody testing [150, 159, 160] including the International Union of Immunology Specialties (IUIS) Autoantibody Standardization Committee (http://asc.dental.ufl.edu/home.html#text), the European Autoimmunity Standardisation Initiative (EASI) [http://www.easi-network.com/], and the Working Group on Harmonization of Autoantibody Tests (WG-HAT) in the framework of the International Federation of Clinical Chemistry and Laboratory Medicine [http://www.ifcc.org/ifcc-scientific-division/sd-working-groups/harmonisation-of-autoantibody-tests-wg-hat/]. Despite significant efforts to standardize autoantibody tests [152, 161] and evidence that these groups are now working more closely together, [21] significant variations still exist.

## 8. Conclusions

- Performance data (including LRs) of the method used to detect ANA and appropriate explanation should be made available to the clinician.
- ANA test results are only a portion of the information that aids in the diagnosis of systemic autoimmune diseases and are an adjunct to the clinician's diagnostic repertoire.
- Both IIF on HEp-2 cells and solid phase immunoassays have their individual advantages and limitations.
- Standardized nomenclature of diseases and associated autoantibodies is an important goal for immediate consideration by advisory groups.

## 9. Future Perspectives

Despite significant evolution and improvements in ANA and related autoantibody testing, including the arrival of novel and promising technologies, several limitations still persist and need to be addressed. First, the terminology and nomenclature used to identify and refer to various autoantibodies need to be standardized. Second, the classification criteria and nomenclature of individual SARD and related autoimmune diseases must continue to evolve and keep abreast of biomarker identification. Third, the corresponding immunoassays and diagnostic platforms used for the various clinical applications need to be based on standardized reference samples of defined specificities. This possibility could include the development and validation of disease specific screening assays (i.e., SLE Screen, SSc Screen) on solid phase technologies. Fourth, a clearly defined strategy needs to be developed to facilitate clinicians and laboratory scientists alike becoming more familiar with and be able to intelligently use objective interpretation of autoantibody results through an understanding of ROCs and LRs. Lastly, diagnostic algorithms need to be adjusted to the clinical and laboratory setting considering the referral pattern, the sample testing volume, and health economic aspects (i.e., reimbursement).

## Figures and Tables

**Table 1:** Statistical terms relevant for ANA testing.

Statistical measure	General explanation	Implication for ANA
Sensitivity	Statistical measure of how accurately a test correctly identifies diseased individuals	ANA is used as screening test. High sensitivity is important. The sensitivity for different AARD varies (i.e., higher in systemic lupus erythematosus versus myositis)

Specificity	Statistical measure of how well a test correctly identifies absence of the disease in question	Importance of specificity depends on pretest probability. In settings with low pretest probability, high specificity is required.

Diagnostic efficiency	Combination of sensitivity and specificity	Not commonly used

False negative (clinically)	Negative test result of a diseased individual	ANA is used as screening test. False negative results are undesirable. However, in all AARD, patients without a positive ANA test exist. Therefore, a negative result should never be used to rule out AARD.

False positive (clinically)	Positive test result of an individual without the disease in question	In case of low pretest probability, false positive results significantly impact the posttest probability

False negative (analytically)	Negative test result in the presence of the respective analyte	See negative positive (clinically)

False positive (analytically)	Positive test result in the absence of the respective analyte	See false positive (clinically)

Positive predictive value	Ratio of true positive to combined true and false positives.	Depends on the prevalence (pretest probability)

Negative predictive value	Ratio of true negatives to combined true and false negatives.	Depends on the prevalence (pretest probability)

Positive likelihood ratio	The probability of a positive test results in patients with the disease divided by the probability of a positive test result in individuals without the disease. Independent from prevalence.	^ #^Important information for clinicians. Should be included in the laboratory report together with an explanation of its significance in the context of the test result.

Negative likelihood ratio	The probability of a negative test result in patients with the disease divided by the probability of a negative test result in individuals without the disease. Independent from prevalence.	^ #^Important information for clinicians. Should be included in the laboratory report together with an explanation of its significance in the context of the test result.

^#^The importance of the likelihood ratio in the laboratory report is controversially discussed, but might improve use of ANA test results in the future.

**Table 2:** Anti-nuclear antibodies (ANA) in different ANA associated autoimmune rheumatic diseases and healthy individuals.

Antibody	AARD					
	SLE	SSc	SjS	IIM	MCTD	HI
dsDNA	40–70%	<3%	<3%	<3%	<3%	<3%
Chromatin	40–70%	<3%	<3%	<3%	5–18%	<3%
RNP	10–40%	5–15%	<3%	5–15%	100%^5^	<3%
Sm	5–20%	<2%	<1%	<1%	<2%^2^	<1%
SS-A/Ro60	40–70%	3–10%	60–90%	<3%	<3%	<3%
Ro52/ TRIM21	40–70%	15–30%	70–90%	25–50%	<3%	<3%
SS-B/La	15–30%	1–5%	60–80%	5–15%	<3%	<3%
Scl-70 (topo I)	0–5%	20–40%^4^	<3%	<3%	<3%	<1%
Jo-1	1–3%	1–3%	<2%	15–30%	<2%	<1%
Centromere	2–5%	20–40%^4^	5–10%	1–3%	2–5%	<3%
RNA Pol III	<1%	5–25%^4^	<1%	<1%	<1%	<1%
Ribosomal P	10–30%	<2%	<2%	<2%	<2%	<1%
PM/Scl	1–3%	5–10%	<2%	5–10%	<2%	<3%
Mi-2	<1%	3–8%	<1%	5–15%^1^	<1%	<3%
Ku	5–20%	3–8%	<3%	3–10%^3^	<3%	<3%
PCNA	<5%	<1%	<1%	<1%	<1%	<3%
Th/To	<1%	3–10%	<1%	<1%	<1%	<1%

^1^Rare in PM, higher prevalence in DM; mild form of disease; early during development.

^2^Prevalence depends if antigen contains SmBB′ (cross-reactive with RNP).

^3^Very high titer in PM.

^4^Anti-Scl-70, anti-centromere, anti-RNA Pol III antibodies tend to be mutually exclusive.

^5^Part of the classification criteria, therefore should be 100%; however, depending on assay used, some patients might be negative.

Note: Prevalence values were established based on literature and consensus of authors.

Abbreviations: DM: dermatomyositis; IIM: idiopathic inflammatory myopathy (polymyositis/dermatomyositis); MCTD: mixed connective tissue disease; PCNA: proliferating cell nuclear antigen; PM: polymyositis; RA: rheumatoid arthritis; RNA pol III: RNA polymerase III; RNP: ribonucleoprotein; Sm: Smith antigens (U2-U6 RNP); SjS: Sjögren's syndrome; SLE: systemic lupus erythematosus; SPA: Solid phase assay; SSc: systemic sclerosis; TRIM: tripartite motif.

**Table 3:** Commercially available immunoassays for ANA testing.

Antigen	Automated screening assays					Multiplex assays			Line immunoassays/dot blots					
	EliA CTD	EliA Symphony	QUANTA Flash ENA7	QUANTA Flash CTD Screen Plus	Alegria ANA Detect	Bio-Plex 2200 ANA ALBIA	AtheNA Multi-Lyte Anti-Nuclear Antibodies (ANA)	FIDIS Connective 10	ANA Profile	ImmcoStripe ANA Advanced	ANA12 IgG BlueDot	ANA-12 Pro (BLOT)	recomLine ANA/ENA IgG	ANA Lia MAX
	Thermo Scientific	Thermo Scientific	INOVA Diagnostics	INOVA Diagnostics	Orgentec	Bio-Rad	Zeus	Theradiag	Euroimmun	IMMCO Diagnostics	D-Tek	AESKU Diagnostics	MIKROGEN Diagnostics	HUMAN Diagnostics
U1-RNP	X	X	X	X		X	X		X		X	X	X	
U1-RNP/Sm		X	X	X	X			X			X			
U1-RNP 68 kDa					X					X				X
U1-RNP A					X					X				X
U1-RNP C					X					X				X
SS-A/Ro60	X	X	X	X	X	X^(i)^	X	X	X	X	X	X	X	X
Ro52/TRIM21	X	X	X	X	X	X^(i)^			X	X		X	X	X
SS-B/La	X	X	X	X	X	X	X	X	X	X	X	X	X	X
Centromere	X	X		X^#^	X	X	X	X		X	X^#^	X	X	X
Scl-70/topo I	X	X	X	X	X	X	X	X	X		X	X	X	X
Jo-1	X	X	X	X	X	X	X	X	X	X	X	X	X	X
Fibrillarin/U3RNP	X													
RNA-Pol III	X			X										
Ribosomal P	X			X		X		X	X	X	X	X	X	X
PM/Scl	X			X					X					X
PM/Scl-75										X				
PM/Scl-100					X					X				
PCNA	X			X					X	X	X		X	X
Mi-2	X			X					X	X				X
Sm	X			X	X	X	X		X	X	X	X	X	X
dsDNA	X			X	X	X		X	X	X		X	X	X
Nucleosome					X				X	X		X		X
Histone					X		X		X	X		X	X	X
Ku				X					X	X	X			X
SRP54										X				
AMA-M2									X	X				X

Note: some companies offer several line immunoassays for ANA detection. Most comprehensive assays from different companies are shown.

AMA: Anti-mitochondrial antibodies; PCNA: Proliferating cell nuclear antigen; SRP: signal recognition particle.

NOTE: RNP and RNP/Sm contain the subunits RNP-A, RNP-C and RNP-68 kDa.

^(i)^Reported outside the United States as Ro60 and Ro52 and as SS-A in the United States.

^#^Contains CENP-A and CENP-B.

**Table 4:** Clinical utility of ANA testing in different diseases.

Diagnosis	Clinical utility	ANA prevalence	Monitoring/prognosis	Comments
SLE	Very useful	90–95%	Not useful	ANA IIF superior to ANA solid phase assays
SSc	Very useful	85–95%	Not useful	ANA IIF superior to ANA solid phase assays
SjS	Useful	50–60%	Not useful	ANA solid phase assays superior to ANA IIF; SS-A reactivity can be missed by ANA HEp-2
AIM	Somewhat useful	50–60%	Not useful	ANA solid phase assays superior to ANA IIF; Jo-1 reactivity can be missed by ANA HEp-2
MCTD	Very useful	90–100%	Not useful	High titer anti-U1-RNP are highly indicative for MCTD
JCA/JIA	Somewhat useful	50–60%	Very useful	Useful for subset that are at risk of developing uveitis
PBC	Very useful	50–80%	Not proven	ANA IIF superior to solid phase assays; Antibodies to SP100, gp210, nucleoporin p62, lamin B receptor and Ro52 /TRIM21. Anti-gp210 reported association with poor prognosis.
RA	Not useful	15–20%	Not useful	Homogeneous and speckled staining are the most common patterns
APS	Not useful	40–70%	Not useful	Might indicate systemic autoimmunity in primary APS patients
AT	Not useful	10–20%	Not useful	Higher in Grave's disease as compared to Hashimoto`s thyroiditis
Cancer and paraneoplastic syndromes	Not useful, or utility not established	20–50%	Not useful	Antibodies to CENP-F and to other proteins might be useful to help in the diagnosis of cancer; p53 has been discussed; not many systematic studies on ANA in cancer
AIH	Useful	40–80%	Not useful	Prevalence depends on phase of the disease

Abbreviations: AIH: autoimmune hepatitis; AIM: autoimmune inflammatory myopathy (polymyositis, dermatomyositis); APS: anti-phospholipid syndrome; AT: autoimmune thyroiditis; JCA/JIA: juvenile chronic arthritis/juvenile inflammatory arthritis; MCTD: mixed connective tissue disease; PBC: primary biliary cirrhosis; RA: rheumatoid arthritis; SjS: Sjögren's syndrome; SLE: systemic lupus erythematosus; SSc: systemic sclerosis NOTE: Prevalence values are based on diagnostic samples (not treated patients).

**Table 5:** Advantages and disadvantages of the HEp-2 ANA test.

Advantages	Disadvantages
Variety of different target autoantigens (>100)	Subjectivity
Some autoantibodies can be identified without confirmatory testing (i.e., anti-centromere)	Poorly standardized across manufacturers
Discovery tool for novel autoantibodies	Requires training and expertise
Useful for a spectrum autoimmune diseases (i.e., autoimmune hepatitis)	Low sensitivity for certain clinically important autoantibodies (i.e., Jo-1, ribosomal P, SS-A/Ro60, Ro52/TRIM21)
	Low specificity (high false positive rate)

**Table 6:** Overview of defined ANA patterns (modified from Wiik et al., 2010 [13]).

Pattern group	Pattern
Nuclear envelope (membrane)	Smooth nuclear envelope
	Punctate nuclear envelope

Nuclear	Homogeneous pattern
	Large speckled
	Coarse speckled
	Fine speckled
	Fine grainy Scl-70-like
	Pleomorphic speckled (i.e., PCNA)
	Centromere
	Multiple nuclear dots
	Coiled bodies (few nuclear dots)
	Dense fine speckled
	Isolated metaphase chromosomes

Nucleolar	Homogeneous nucleolar
	Clumpy nucleolar
	Punctate nucleolar

Mitotic spindle apparatus	Centriole (centrosome)
	Spindle pole (NuMa) (MSA-1) (HSeg5)
	Spindle fiber
	Midbody (MSA-2)
	CENP-F (MSA-3)

Cytoplasmic	Diffuse
	Fine speckled
	Mitochondrial
	Discrete dots: GW bodies, endosomes, lysosomes
	Golgi complex
	Intercellular contact proteins
	Fibers and cytoskeleton
	Rods and rings

Negative	

**Table 7:** Automated digital ANA reading systems.

Instrument	NOVA View	AKLIDES	EUROPattern	Image Navigator	Helios	ZENIT G Sight
Manufacturer	INOVA diagnostics	Medipan	Euroimmun	Immunoconcepts	Aesku	Menarini

LIMS connection (software)	Yes (QUANTA Link)	Yes (system independent, standard XML interface)	Yes (EUROLabOffice)	Yes (direct) Optional: lab traffic control	Yes (direct) Optional: Aesku.Lab middleware	Yes (ZenIT)

Slide identification via barcode	Yes by handheld scanner	Yes by handheld scanner	Yes by integrated scanner	Yes	Yes by integrated scanner	Yes by integrated scanner

Loading capacity	5 slides (up to 60 wells)	5 slides (up to 60 wells)	50 slides (up to 500 wells)	4 slides (up to 84 wells)	20 slides (up to 240 wells)	5 slides (up to 70 wells)

Image acquisition speed	~45 s/well for 3 images	~40 s/well	<20 s/well	~25 s/well for 4 images	10 s/picture Customizable from 1 to 10 images	>60 s/well number of pictures: 5 (small scan), 50 (medium scan), or 220 (full scan)

100% QC for substrate and process integrity/counterstaining	Yes/DAPI	Yes/DAPI	Yes/Propidium iodide	None/None	None/None	None/None

Automatic pos./neg. discrimination incl. presorting of images	Yes	Yes	Yes	Yes	Yes	Yes

Batchwise verification of negative samples	Yes	Yes	Yes	Yes	Yes	Yes

Automatic pattern recognition	Yes	Yes	Yes	No	No	Yes

Pattern Analysis method	Pattern recognition by mathematical algorithm	Pattern recognition by mathematical algorithm	Pattern recognition by mathematical algorithm	No pattern matching capabilities	No pattern matching capabilities	Pattern recognition by mathematical algorithm

Number of recognizable ANA staining pattern list out	6 Homogeneous Speckled Centromere Nucleolar Nuclear dot Cytoplasm Negative Positive unrecognized	10 Homogeneous Speckled Centromere Nucleolar Nuclear dot Cytoplasm Negative Positive unrecognized	8 Homogeneous Speckled Centromere Nucleolar Nuclear dot Cytoplasm Negative Nuclear rim Mixed pattern mitotic	None	None	5 Homogeneous Speckled Centromere Nucleolar Cytoplasm Negative

Analysis of mixed staining pattern	Limited (homogeneous/ nucleolar)	Yes	Yes	No	No	No

Merged results per patient (different dilutions)	Yes	Yes	Yes	Yes	Yes	Yes

Final result validation possible while system processes remaining samples	Yes	No	Yes	Yes	Yes	No

Instrument calibration to minimize variability	Yes	Yes	Yes	Yes	Yes	No

Integration with slide processing in 1 instrument	No	No	No	No	Yes	No
